# Anaphylaxis to husband's seminal plasma and treatment by local desensitization

**DOI:** 10.1186/1476-7961-6-13

**Published:** 2008-12-05

**Authors:** Jaechun Lee, Sohyung Kim, Miok Kim, Young-Bae Chung, Jung-Sik Huh, Chul Min Park, Keun Hwa Lee, Jeong Hong Kim

**Affiliations:** 1 Department of Medicine, School of Medicine, Jeju National University, Jeju, Korea; 2 Department of Parasitology, School of Medicine, Jeju National University, Jeju, Korea; 3 Department of Urology, School of Medicine, Jeju National University, Jeju, Korea; 4 Department of OB & GYN, School of Medicine, Jeju National University, Jeju, Korea; 5 Department of Microbiology, School of Medicine, Jeju National University, Jeju, Korea; 6 Department of Otorhinolaryngology, School of Medicine, Jeju National University, Jeju, Korea

## Abstract

Hypersensitivity to human seminal fluid is rare but can be life threatening. We report a case of IgE-mediated anaphylaxis to seminal plasma that was diagnosed by skin prick tests and successfully treated by local desensitization. A 32-year-old woman suffering from angioedema and hypotension after exposure to semen was treated with epinephrine upon admission. Skin prick tests and immunoblotting for IgE binding components showed that she was sensitized to her husband's seminal plasma. Local desensitization, which persisted for six months, was achieved by intravaginal administration of serial dilutions of her husband's seminal plasma.

## Introduction

Hypersensitivity to human seminal fluid is rare. The clinical features of this condition are diverse and vary from weak reactions such as vaginal itching after coitus to systemic reactions such as systemic urticaria or even anaphylactic shock, which can be life-threatening [1,2]. Diagnosis is usually based on medical history. Symptoms usually occur immediately after exposure to semen or within an hour of coitus [3]. This condition should be distinguished from hypersensitivity to latex, spermicidal agents, or lubricants.

## Case presentation

A 32-year-old woman was admitted to our emergency department because of an abrupt onset of hives characterized by periorbital redness and swelling, breathlessness, and wheezing. Angioedema and hypotension (90/60 mmHg) were observed. She was resuscitated by administration of subcutaneous epinephrine, intravenous corticosteroid, and antihistamine.

The patient was married and had delivered a baby four months previously. After abstinence from sexual intercourse for several months before and after the delivery, she had intercourse with her husband. After coitus, during which intravaginal ejaculation occurred, she experienced an itching sensation of the perineum and swelling of the vulva, which subsided within a few hours without treatment. Her gynecologist diagnosed nonspecific vaginitis, but treatment did not improve her condition. Several days before she presented at our emergency room, she experienced an episode of itchy hives at sites on her trunk that had been exposed to semen. The hives subsided the next morning. On the day of her visit to the emergency room, she suffered from an itchy, swollen vulva and breathlessness after coitus. She reported no extramarital sexual activity. She had no history of allergy and had never used contraceptives or contraceptive devices.

**Figure 1:**
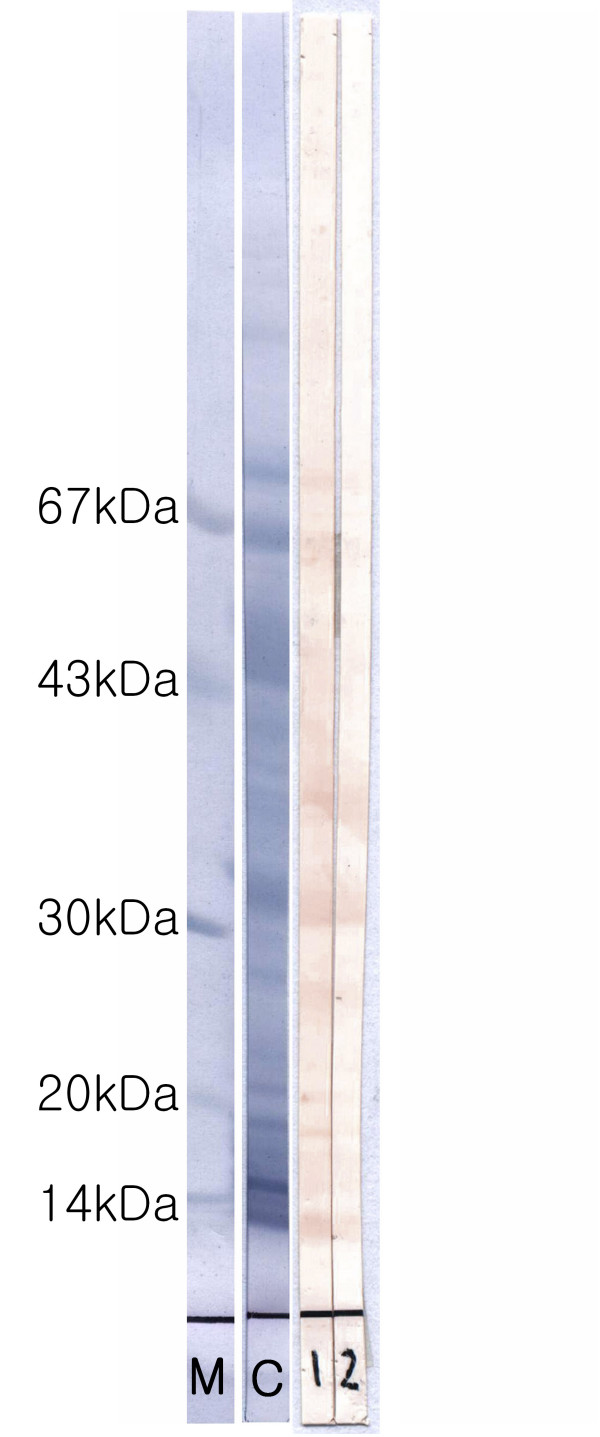
**The results of SDS-PAGE and IgE-immunoblot with the husband's seminal plasma**. M: marker protein, C: Coumacie blue staing of SDS-PAGE, 1 and 2: IgE-immunoblot with patient's serum (1:1 & 1:10 dilution with PBS, respectively).

After four weeks' abstinence from intercourse, skin prick tests were performed using 40 common food allergens and 55 common inhalant allergens. All tests were negative. Samples of her husband's semen and blood were also used for skin prick tests. Seminal plasma was separated by centrifugation of semen at 3000 rpm for 30 min. Serial dilutions of samples in 0.9% saline were used for the skin prick tests. Skin prick tests with dilutions of her husband's seminal plasma resulted in a 2 mm × 2 mm wheal at a 1:100 dilution and a 6 mm × 5 mm wheal at a 1:10 dilution. Skin prick tests with her husband's serum were negative. The positive control (1 mg/mL histamine) resulted in a 5 mm × 5 mm wheal and the negative control (0.9% saline) did not induce any reaction. Specific IgE and their binding components from seminal plasma protein of patient's husband were detected by sodium dodecyl sulfate polyacrylamide gel electrophoresis with immunochemical staining (Figure 1).

The patient was diagnosed with hypersensitivity to seminal plasma on the basis of her history, the results of skin prick tests, and the presence of IgE-specific binding activity in her husband's seminal plasma.

Although the use of condoms and self-injectable epinephrine would normally have been recommended, this patient expressed a desire to conceive a second baby. As she wanted conception to occur as a result of sexual intercourse with her husband, local desensitization with serial diluted seminal plasma was performed as described previously [4,5]. Briefly, tenfold serial dilutions of seminal plasma with normal saline (1:1 to 1:10000) were prepared. Antihistamine was injected as a premedication. While the patient's vital signs were monitored, 1 mL aliquots of diluted seminal plasma were inserted into the vagina at 45 min intervals. She complained of local but tolerable itching when the 1:100 dilution was administered. No systemic reactions were observed, even when pure seminal plasma was administered. She also experienced no reaction after coitus the following day. We recommended that she have sexual intercourse more than once weekly to maintain the desensitization and that she use self-injectable epinephrine if necessary. She did not experience any symptoms during the six-month medical observation period.

## Discussion

Hypersensitivity to human seminal plasma in women is an exceptionally rare phenomenon. Through the repetitive exposure to foreign protein, that is seminal plasma, to which the victim is sensitized, the diverse immediate type hypersensitivity reaction happens during or soon after coitus. It ranged from local itchy sense to systemic anaphylactic shock or both, which happens during coitus or within 30 min after coitus [6].

The treatment is complete avoidance if available and acceptable, but it happens in sexually active fertile females. We face the worst situation when the patient wants to be pregnant soon. Pregnancy could be successfully and safely achievable by artificial insemination [7]. Successful pregnancies even through intercourse were reported after intravaginal desensitization [4,8].

In this case, patient suffered from local hives to systemic reaction with progressive manner. Without systemic reaction, it might be neglected even by gynecologists, because it mostly happens during or within 30 min after exposure and the hives usually disappear in a few hours. If suspected, an intravaginal provocation test with seminal plasma will facilitate definitive diagnosis, but the skin prick test is safer and enables a diagnosis to be made when a systemic reaction is indicated.

This case of anaphylaxis to seminal plasma was successfully treated by local desensitization with seminal plasma, done by patient's request for pregnancy through natural coitus with her husband. If repetitive exposures are unavoidable, local desensitization and maintenance thereof should be considered.

## Consent

Written informed consent was obtained from the patient for publication of this case report and accompanying images.

## Competing interests

The authors declare that they have no competing interests.

## Authors' contributions

JL and SK firstly found the case and carried out the clinical tests. JL, MK and JHK drafted the manuscript. CMP and JSH designed and carried out the desensitization procedure. YBC and KHL carried out immunoblot tests. All the authors read and approved the final manuscript.
